# Study of Otorhinolaryngological Manifestations in Symptomatic COVID-19-Positive Patients at Tertiary Health Care Hospital: A Cross-sectional Study

**DOI:** 10.1055/s-0044-1786831

**Published:** 2024-07-05

**Authors:** Disha Amar Methwani, Nitin Deosthale, Sonali Khadakkar, Kanchan Dhote, Vivek Harkare

**Affiliations:** 1 Department of ENT, N. K. P. Salve Institute of Medical Sciences & Research Centre and Lata Mangeshkar Hospital, Nagpur, Maharashtra, India

**Keywords:** COVID-19, otorhinolaryngology manifestation, anosmia, headache, loss of taste

## Abstract

**Introduction** The coronavirus disease 2019 (COVID-19), also referred to as the 2019 novel coronavirus, is caused by a single-stranded positive-sense RNA virus. This infectious agent spreads through respiratory routes, primarily utilizing aerosols. In our study, we shed light on ear, nose, and throat (ENT) manifestations, which can be considered as prognostic clinical biomarkers to reduce forthcoming complications among such critically ill patients. This makes it feasible for researchers to investigate or fetch early diagnosis in such cases with ease without the use of large, extensive hospital-base setups.

**Objective** To study the otorhinolaryngological (ENT) manifestations in symptomatic COVID-19 patients.

**Methods** From January to December 2021, a cross-sectional observational study was conducted at the Department of ENT of a tertiary care hospital in central India. All eligible symptomatic COVID-19 patients admitted to this institution during the study period were consecutively enrolled. The Institutional Ethics Committee gave its permission for the project.

**Results** Out of the total of 1,375 patients, 78% of the patients diagnosed with COVID exhibited symptoms related to the ENT, while the remaining 22% did not display any ENT manifestations. Anosmia (71.2%), sore throat (55.62%), headache (44.3%), and loss of taste (66.3%) were among the most common ENT symptoms.

**Conclusion** The present study highlights ENT manifestations, which play a crucial role in the early diagnosis of COVID-19 patients, ensuring faster treatment and isolation for better containment of the disease. Symptomatic treatment has shown efficacy, but objective tests are needed to prevent overestimation, understand pathogenesis, and enhance treatment.

## Introduction

Coronavirus disease 2019 (COVID-19), a disease caused by the severe acute respiratory syndrome corona virus (SARS-CoV-2) single-stranded RNA virus, emerged in December 2019 in Wuhan, Hubei province, China. Initially presenting as a pneumonia-like illness with an unknown source, 1 2 the World Health Organization (WHO) officially declared COVID-19 as a pandemic and a global public health emergency of international concern on January 30, 2020. 1 3

The increasing incidence has underscored the vulnerability of the nasal cavity to SARS-CoV-2 disease. Comparative analyses of the pathology and virology of SARS-CoV, SARS-CoV-2, and MERS-CoV have been conducted by scientists. Extensive research has confirmed that different pathogenic corona viruses have specific target sites within the body. Severe acute respiratory syndrome coronavirus 2 primarily affects the nose and throat, whereas SARS-CoV primarily targets the lungs and Middle East respiratory syndrome coronavirus (MERS-CoV) affects type-2 pneumocytes. Notably, viral loads in the nasal cavity were found to be higher than in the pharynx for both symptomatic and asymptomatic individuals, suggesting that the nasal cavity serves as the primary entry point in the early stages of disease. 4 5 The symptoms of COVID-19 closely resemble those of the seasonal flu, including fever, cough, shortness of breath, fatigue, muscle aches, sore throat, head pain, and loss of taste or smell. 6 Coronavirus disease 2019 can manifest a wide spectrum of symptoms, ranging from asymptomatic or mild manifestations to severe illness and, in some cases, even mortality. 7 Ear, nose, and throat (ENT) symptoms are commonly observed in COVID-19, particularly in individuals with mild-to-moderate illness. 8

Due to its significant capacity for human-to-human transmission, a global pandemic emerged, originating in Assam, where the initial case was recorded on March 31, 2020. Potential modes of transmission for this virus include contact, respiratory droplets, airborne particles, contaminated surfaces (fomites), and the fecal-oral route, blood-borne transmission, mother-to-child transmission, and zoonotic transmission from animals to humans. 9 The symptoms of COVID-19 in otorhinolaryngology (ORL) and the increased risk of related consequences have not been extensively explored in the published literature. Hence, spreading awareness of the above-stated ORL signs and symptoms is essential. During this devastating COVID-19 wave, these ENT manifestations, which include the ENT can serve as important biomarkers to reduce the forthcoming complications among COVID-19 patients.

Hence, this study aimed to identify and analyze the various manifestations related to the ENT in patients who have tested positive for COVID-19 and are exhibiting symptoms.

## Methods

This captivating observational study was conducted at the prestigious Department of ENT of a tertiary care hospital in central India. Spanning from January to December 2021, this hospital-based cross-sectional study aimed to shed light on valuable insights regarding the subject at hand. Every consecutive COVID-19 patient who was admitted to this institute for the period of the study and who fit into the inclusion criteria were enlisted for the study. The Institutional Ethics Committee provided its ethical approval (ECR/88/Inst/MH/2013/RR-19).

### Inclusion Criteria

1. Reverse transcriptase-polymerase chain reaction (RT-PCR) COVID test/rapid antigen test (RAT) positive symptomatic patients of either gender and age admitted in an isolation ward of N.K.P. Salve Institute of Medical Sciences & Research Centre and Lata Mangeshkar Hospital in the given time frame.
2. Those who consented to take part in the study.

### Exclusion Criteria

1. History of similar ENT manifestations before COVID-19 positive status.
2. Very critical patients who are unable to give a complete history.
3. Patients with a previous medical history of oral or nasal cavity surgery or radiotherapy were excluded from the study.
4. Patients with a history of mental illnesses.

### Patient Enrollment Protocol

Throughout the study period, a total of 1,827 cases were admitted to the hospital, of which 260 patients were critically ill and succumbed to the disease. Ultimately, 1,375 patients met the inclusion criteria for the study. Unfortunately, 192 patients either did not meet the selection criteria or their records were not available for analysis. The medical team diligently recorded a comprehensive history for each of the 1,375 patients, and conducted vital studies as feasible, carefully documenting the findings in a well-structured proforma.

## Observations and Results

Among the patients, the majority were male, with 68%, while women represented only 32%. Regarding age distribution, the highest proportion of patients was observed in the 46-to-55 years age group (31.71%), followed by the 36-to-45 years age group (19.78%). The lowest representation was observed in the > 75 years age group (4.07%).

A total of 1,375 patients were included in the analysis, out of which 1,075 (78.18%) exhibited ENT manifestations, while 303 (22.04%) did not. ( Table 1 )

**Table 1:** Demographic distribution of patients

Sex distribution	No. of cases	Percentage
**Male**	937	68%
**Female**	438	32%
**Age distribution**		
**< 25 Years**	91	6.62%
**26–35 years**	183	13.31%
**36–45 years**	272	19.78%
**46–55 years**	436	31.71%
**56–65 years**	145	10.55%
**66–75 years**	192	13.96%
**> 75 years**	56	4.07%
**Total patients**	1,375	100.00%
**with ENT manifestation**	1,075	78.18%
**without ENT manifestation**	303	22.04%

Abbreviation: ENT, ear, nose, and throat.

Among the non-ENT clinical manifestations, fever was the most common symptom, observed in 1,051 cases (76%). Cough was reported in 271 cases (20%), while breathlessness was present in 267 cases (19%). Diarrhea was experienced by 84 patients (6%), and nausea/vomiting was reported by 47 patients (3%). Malaise/body ache was a prevalent symptom, affecting 636 patients (46%). Chest pain and hemoptysis were relatively rare, with 9 cases (1%) and 7 cases (1%), respectively. Loss of appetite was noted in 935 patients (68%). ( Table 2 )

**Table 2:** Distribution of patients with common non-ENT clinical manifestation of COVID-19

Clinical feature	No. of cases	Percentage
**Fever**	1051	76%
**Cough**	271	20%
**Breathlessness**	267	19%
**Diarrhea**	84	6%
**Nausea/vomiting**	47	3%
**Malaise/bodyache**	636	46%
**Chest pain**	9	1%
**Hemoptysis**	7	1%
**Loss of appetite**	935	68%

Abbreviations: COVID-19, coronavirus disease 2019; ENT, ear, nose, and throat.

* Multiple observations allocated.

An analysis of COVID-19 patients revealed distinct nasal and sinus manifestations. Anosmia/hyposmia, characterized by a loss or reduction in the sense of smell, was the most prevalent symptom, affecting 979 cases (71.20%). Nasal discharge was reported in 165 cases (12.00%), while nasal blocking/congestion was present in 250 cases (18.18%). A smaller subset of patients experienced nasal bleeding/epistaxis, with 55 cases (4.00%) reported. Notably, 609 patients (44.29%) experienced headache/facial pain, which may be linked to sinus involvement. These findings shed light on the diverse nasal and sinus symptoms observed in COVID-19 patients, emphasizing the prominence of anosmia/hyposmia and the significance of headache/facial pain. ( Table 3 )

**Table 3:** Distribution of patients with Nasal& sinus manifestations of COVID-19

Clinical features	No. of cases	Percentage
**Anosmia/hyposmia**	979	71.20%
**Nasal discharge**	165	12.00%
**Nasal blocking/congestion**	250	18.18%
**Nasal bleeding/epistaxis**	55	4.00%
**Headache/facial pain**	609	44.29%

Abbreviation: COVID-19, coronavirus disease 2019.

Among the oral cavity and throat manifestations observed in COVID-19 patients, sore throat was the most common symptom, affecting 765 cases (55.64%). Loss of taste or taste disturbance was reported by 912 patients (66.33%). Oral lesions and crusting were observed in 89 cases (6.47%). Heartburn or regurgitation, indicative of acid reflux, was experienced by 67 patients (4.87%). Difficulty in swallowing was reported in 47 cases (3.42%). ( Table 4 )

**Table 4:** Distribution of patients with oral cavity and throat manifestation of COVID-19

Symptoms	No. of cases	Percentage
**Sore throat**	765	55.64%
**Loss of taste/taste disturbance**	912	66.33%
**Oral lesions & crusting**	89	6.47%
**Chest burn/regurgitation**	67	4.87%
**Difficulty in swallowing**	47	3.42%

Abbreviation: COVID-19, coronavirus disease 2019.

An analysis of COVID-19 patients revealed various manifestations affecting the ear. Among these, ear blocking/ear pain was reported in 22 cases (1.60%). Tinnitus, characterized by the perception of noise or ringing in the ears, was experienced by 14 patients (1.02%). Additionally, a small subset of patients reported giddiness, with 8 cases (0.58%). Furthermore, 4 patients (0.29%) reported hearing loss. ( Table 5 )

**Table 5:** Distribution of patients with ear manifestations of COVID-19

Symptoms	No. of cases	Percentage
**Ear blocking / ear pain**	22	1.60%
**Tinnitus**	14	1.02%
**Giddiness**	8	0.58%
**Hearing loss**	4	0.29%

Abbreviation: COVID-19, coronavirus disease 2019.

A comprehensive examination of COVID-19 patients uncovered several atypical ENT diagnoses. Within this group, cervical lymphadenopathy, which manifests as enlarged lymph nodes in the neck, was identified in 25 cases (1.8%). Parotitis, an inflammation of the parotid glands, was observed in 4 cases (0.3%). Moreover, subacute thyroiditis, characterized by inflammation of the thyroid gland, was diagnosed in 3 cases (0.2%). Facial nerve palsy, resulting in facial muscle weakness, was noted in 3 cases (0.2%). Acute suppurative otitis media (ASOM), an infection affecting the middle ear, was detected in 2 cases (0.15%). Two cases (0.15%) experienced sudden sensorineural hearing loss (SNHL), and a rapid decline in auditory function. Additionally, 7 cases (0.5%) were afflicted with mucormycosis, a fungal infection. ( Table 6 )

**Table 6:** Frequency of unusual ENT diagnosis in COVID-19 patients

Diagnosis	No. of cases	Percentage
**Cervical lymphadenopathy**	25	1.8%
**Parotitis**	4	0.3%
**Subacute thyroiditis**	3	0.2%
**Facial nerve palsy**	3	0.2%
**ASOM**	2	0.15%
**Sudden SNHL**	2	0.15%
Mucormycosis*	7	0.5%

Abbreviations: COVID-19, coronavirus disease 2019; ENT, ear, nose, and throat; SNHL, sensorineural hearing loss.

* While patients were admitted in COVID ward/ICU.

## Discussion

Coronavirus disease 2019 presents a wide range of clinical manifestations, spanning from asymptomatic cases to severe complications such as septic shock as well as multi-organ dysfunction. Despite its rapid global spread, the clinical features of COVID-19 remain largely ambiguous. Previous studies have highlighted the need for further understanding and characterization of this disease. 10 11 Research suggests that COVID cases are more likely to experience non- ENT symptoms, such as fever and cough, than ENT manifestations. Nevertheless, it is worth noting that ENT symptoms are more frequently observed during the initial phases of the infection. 12 Recognizing these ENT symptoms can assist in identifying and isolating individuals who have mild symptoms (pauci-symptomatic) of COVID-19. 13

These comparisons provide insights into the consistency or variations in ENT manifestations observed across different studies on COVID-19 patients ( Table 7 ). The COVID-19 virus can impact individuals of any age, gender, socioeconomic status, or religious background. Our study reflected a similar pattern, as patients across all age groups were affected, with a higher incidence observed among individuals aged 20 to 40 years. 14 Similarly, in the present study, the sample consisted of 68% (937) male and 32% (438) female patients. The age distribution included 6.6% (91) below 25 years, 6% (183) between 26 and 35 years, 19.7% (272) between 36 and 45 years, 31.7% (436) between 46 and 55 years, 10.5% (145) between 56 and 65 years, 14% (192) between 66 and 75 years, and 4% (56) above 75 years old.

**Table 7:** Comparison of ENT manifestations in COVID-19 patients

ENT Manifestations	Current Study (%)	El Anwar et al. 15 (%)	Smitha, S.G., 14	Alrusayyis, Danah et al. 19 (%)	Savtale, Saee et al. 20 (%)	Mahmoud, M.S., 21	Özçelik Korkmaz 22
**Anosmia/hyposmia**	55.62	6	13.4	–	55.5	42.3	37.9
**Sore throat**	55.64	11.3	21	12.45	47.22	45.8	32.7
**Headache/facial pain**	44.29	10.7	25.6	22.96%	37.77	42	37
**Loss of taste**	66.33	6	16.6	68.09	58.88		41.3
**Nasal discharge**	12.00	2.1	21.8	14.01	21.11	19.5	13.7
**Nasal blocking**	18.18	4.1		1.17	–		27.5
**Tinnitus**	1.02	–	1.1	–	66.66		11.2
**Giddiness**	0.58	–	7.1	1.56	–		31.8
**Hearing loss**	0.29	–	0.3	–	54.44	0.6	5.2

Abbreviations: COVID-19, coronavirus disease 2019; ENT, ear, nose, and throat.

Within our study, we observed that 78% of COVID patients exhibited ENT symptoms, while 22% did not present with any ENT manifestations. Among the patients with ENT symptoms, the most prevalent manifestations were anosmia (55.62%), sore throat (55.62%), headache (44.3%), and loss of taste (66.3%). The study conducted by El-Anwar et al. 15 reported similar findings, with sore throat (13.3%) being the most common ENT manifestation, followed by headache (10.7%), pharyngeal erythema (5.3%), and nasal congestion (4%) among others. Consistent results were also observed in separate studies conducted by Prabhu et al. 16 as well as Chaurasia et al., 17 in whose studies sore throat was identified as the predominant ENT symptom. While the literature mentions stridor and hoarseness as fewer common symptoms, 18 our study did not report any instances of these particular symptoms in the patients.

Symptomatic and supportive treatment were administered to all patients presenting with ENT manifestations. The majority of patients experienced a resolution of these symptoms within a span of 20 days. However, a subset of patients (28%) reported persisting symptoms even after recovering from the primary illness. Due to the global pandemic situation, comprehensive follow-up and definitive tests could not be conducted for all patients. Based on research findings, hyposmia (reduced sense of smell) and dysgeusia (altered sense of taste) have been identified as early symptoms occurring within the first 5 to 7 days in COVID-19 patients. 13 It is suggested to quarantine individuals presenting with these symptoms for further testing as they are potential COVID-19 carriers. 18 This approach can aid in the early identification of cases and contribute to breaking the chain of transmission. To the best of our knowledge, the present study was performed to identify specific clinical biomarkers associated with otolaryngological symptoms in COVID-19 patients. This could aid in the development of diagnostic tools and help distinguish COVID-19-related otolaryngological manifestations from other respiratory infections.

The following are some limitations of the present study:

- Collecting and reviewing the data was difficult due to the large COVID-19 health emergency.
- Inadequate incident registration without universal knowledge posed a huge hurdle.
- Patients with mild or asymptomatic disease who were in home isolation were missed from the analysis, which could have altered the current data.
- Endoscopies and other tests were performed in limited cases to avoid exposure to the virus during the pandemic.
- Due to limitations, comprehensive objective studies assessing the extent of loss of smell and taste could not be conducted.

## Conclusion

In diagnosing COVID-19, it is crucial to consider the presence of ENT manifestations. Symptomatic treatment has shown effectiveness in the majority of cases. However, it is imperative to conduct additional studies utilizing objective tests to accurately assess otorhinolaryngological manifestations. Ear, nose, and throat specialists are at a higher risk of contracting COVID-19 as they deal with the upper respiratory tract during consultations, clinical examinations, sample collection, and surgeries.

This study illuminates ENT manifestations as a prognostic biomarker to reduce future complications not only among COVID-19 patients but also among otolaryngologists, as COVID-19 patients frequently come into contact with ENT specialists. Therefore, we recommend avoiding overestimating the number of cases, understanding the underlying mechanisms of these symptoms, improving current treatment protocols, and developing specific treatments. Furthermore, it is crucial to conduct further evaluations of recovered patients to identify any potential long-term consequences of the disease.
